# Síndrome Coronariana Crônica no Brasil: É Preciso Conhecer Mais

**DOI:** 10.36660/abc.20230723

**Published:** 2023-12-14

**Authors:** Adriana Lopes Latado, Julio Cesar Vieira Braga

**Affiliations:** 1 Universidade Federal da Bahia Faculdade de Medicina da Bahia Salvador BA Brasil Universidade Federal da Bahia – Faculdade de Medicina da Bahia, Salvador, BA – Brasil; 2 Universidade Federal da Bahia Hospital Universitário Professor Edgard Santos EBSERH Salvador BA Brasil Universidade Federal da Bahia – Hospital Universitário Professor Edgard Santos/EBSERH, Salvador, BA – Brasil

**Keywords:** Doenças Cardiovasculares, Doença da Artéria Coronária, Prognóstico, Síndrome Coronária Aguda, Epidemiologia, Mortalidade, Envelhecimento

A síndrome coronariana crônica (SCC) é um grupo heterogêneo de enfermidades que engloba a doença aterosclerótica coronariana obstrutiva e não obstrutiva com ou sem infarto do miocárdio (IM) prévio ou revascularização coronariana, além da doença diagnosticada apenas por testes não invasivos.^1^ Segundo dados do *Global Burden of Disease (GBD) Study*, o total de pessoas vivendo com a doença isquêmica do coração no Brasil, sintomática ou não, aumentou de 1,48 milhões para mais de 4 milhões, entre os anos 1990 e 2019.^2^ O crescimento e envelhecimento populacionais e o aumento no diagnóstico são as principais justificativas para esse fenômeno.^2,3^

Embora mantendo-se como principal causa de óbito, a mortalidade pela doença isquêmica do coração tem diminuído nas últimas três décadas em praticamente todo o Brasil, ainda que algumas variações regionais sejam descritas.^2-4^ Homens são mais afetados do que mulheres, e se descreve uma relação inversa entre índice de mortalidade por doença isquêmica do coração e nível educacional ou econômico das regiões.^3,5^ Apesar da existência crescente de dados nacionais acerca da doença isquêmica do coração, coortes regionais ou locais para avaliação de seu prognóstico a longo prazo são escassas, ainda mais se considerarmos a necessidade de frequentes atualizações frente às estratégias de tratamento emergentes.

No artigo Acompanhamento de Dois Anos de Pacientes com Cardiopatia Isquêmica Crônica em um Centro Especializado no Brasil desta edição,^6^ os autores descrevem o perfil clínico-demográfico e o prognóstico de pacientes portadores de doenças cardíacas isquêmicas crônicas (DCIC), acompanhados em um centro terciário de assistência cardiológica, o Instituto do Coração (InCor), em São Paulo, Brasil. O registro foi prospectivo, recrutou participantes entre 2016 e 2018, e teve tempo mediano de seguimento de 2,4 anos. Foram incluídos 625 pacientes, porém apenas 533 tiveram a avaliação do seguimento. A amostra tinha idade mediana de 66 anos, um terço de mulheres e alta prevalência de morbidades, incluindo 87% de eventos/procedimentos cardiovasculares prévios. O estudo revelou uma pequena melhora no controle lipídico no seguimento, em especial, no LDL colesterol, porém sem uma aparente correspondência com a terapia prescrita, uma vez que estatinas tiveram proporção similar de prescrição nos momentos final e basal. Os autores chamam atenção para a baixa prescrição de agente hipolipemiante adicional. Entretanto, não há descrição da frequência de utilização de estatina potente em dose máxima, recomendável antes de associar outros medicamentos. Nesse ponto, também seria importante descrever a prática local, pois o Sistema Único de Saúde fornece sinvastatina gratuitamente, mas atorvastatina apenas através de um burocrático processo, o que limita sua utilização. Em um serviço público no mesmo estado, apenas 3% das prescrições ambulatoriais eram de atorvastatina 80mg/d.^7^

A interpretação desses achados é limitada. O estudo avaliou os participantes no momento basal e a cada ano do seguimento, preferencialmente em visitas presenciais, porém sem a garantia destas. Além disso, houve uma perda de seguimento elevada (11,5%) e não há descrição se esta esteve associada aos fatores de risco, o que pode comprometer a confiabilidade de informações. Além disso, prescrição não significa adesão ao tratamento.

Em relação aos desfechos clínicos, Moreira et al.,^6^ encontraram uma incidência de 7% de morte, IM ou AVE (desfecho primário), ao final do seguimento. Esse achado deve ser comparado aos resultados de outras coortes e gerar reflexões, embora diferenças metodológicas e variações regionais possam justificar diferenças na incidência de desfechos. O registro multicêntrico internacional CLARIFY^8^ encontrou 8% de morte cardiovascular ou IM (desfecho primário) em seguimento de 5 anos de pacientes com SCC. O desfecho secundário, composto de morte cardiovascular, IM não fatal e AVE não fatal, mais próximo do desfecho primário do estudo de Moreira et al.^6^, ocorreu em 9,5%. A coorte CLARIFY avaliou mais que 30.000 indivíduos em 45 países de diversos níveis socioeconômicos. Além de tempo de seguimento diferente, houve adjudicação do desfecho primário no estudo CLARIFY.

No registro brasileiro REACT (2021), 5076 pacientes foram seguidos por um ano, dos quais dois terços estavam em prevenção secundária. A incidência de eventos cardiovasculares ateroscleróticos fatais e não fatais foi estimada em 5,46 por 100 pacientes-ano.^9^ O perfil da amostra revelou semelhanças com o estudo,^6^ porém comparações ficam dificultadas pelas diferenças metodológicas entre os estudos.

Outro achado interessante do estudo^6^ foi a redução de angina ao longo do seguimento de 2,4 anos, com aumento do percentual de pacientes assintomáticos na avaliação final,^6^ Esse dado também foi encontrado no registro CLARIFY, ao avaliar 7212 indivíduos com SCC e angina de peito, os quais, submetidos basicamente à terapia médica otimizada, apresentaram redução de sintomas anginosos de 40% em um ano.^10^ A ausência ou melhora da angina se associou à menor incidência de desfechos cardiovasculares maiores, como morte de causa cardiovascular e IM não fatal,^10^ o que subsidia as recomendações de terapia farmacológica otimizada como estratégia primordial em pacientes com SCC.^11^

O estudo^6^ tem limitações que estão relacionadas à amostragem por conveniência, tamanho amostral e tempo de seguimento relativamente pequenos e estatística inferencial eminentemente exploratória. A perda de seguimento foi alta e a falta de adjudicação dos eventos pode reduzir a validade interna. No entanto, as informações do estudo são importantes, contemporâneas e úteis na caracterização dos pacientes com SCC e seu prognóstico a médio prazo. Sinaliza a necessidade de estudos semelhantes a nível regional ou pesquisas multicêntricas nacionais, de modo de obter informação sobre a efetividade de terapias e a evolução clínica dos portadores de SCC.
