# Supplementary material for: Síndrome Coronariana Crônica no Brasil: É Preciso Conhecer Mais
Source: Arq Bras Cardiol. 2023 Dec 14;120(10):e20230723. [Article in Portuguese] doi: 10.36660/abc.20230723 (PMC10763635; doi:10.36660/abc.20230723)
Supplement: Translation

# Translation: Chronic Coronary Syndrome In Brazil: We Need To Know More

**Authors:** Adriana Lopes Latado, Julio Cesar Vieira Braga

**Affiliations:** 1 Brazil Universidade Federal da Bahia – Faculdade de Medicina da Bahia, Salvador, BA – Brazil; 2 Brazil Universidade Federal da Bahia – Hospital Universitário Professor Edgard Santos/EBSERH, Salvador, BA – Brazil

**Keywords:** Cardiovascular Diseases, Coronary Artery Disease, Prognosis, Syndrome Acute Coronary, Epidemiology, Mortality, Aging

Chronic coronary syndrome (CCS) is a heterogeneous group of diseases encompassing obstructive and non-obstructive coronary atherosclerotic disease with or without previous myocardial infarction (MI) or coronary revascularization, in addition to the disease diagnosed only by non-invasive tests.^1^ According to data from the Global Burden of Disease (GBD) Study, the total number of people living with ischemic heart disease in Brazil, whether symptomatic or not, increased from 1.48 million to more than 4 million between 1990 and 2019.^2^ Population growth, aging, and the increase in diagnosis are the main justifications for this phenomenon.^2,3^

Although remaining the main cause of death, mortality from ischemic heart disease has decreased over the last three decades in practically all of Brazil, although some regional variations have been described.^2-4^ Men are more affected than women, and an inverse relationship is described between the mortality rate from ischemic heart disease and the educational or economic level of the regions.^3,5^ Despite the growing existence of national data on ischemic heart disease, regional or local cohorts to assess its long-term prognosis are scarce, even more so if we consider the need for frequent updates regarding emerging treatment strategies.

In the article Two-Year Follow-Up of Chronic Ischemic Heart Disease Patients in a Specialized Center in Brazil of this edition,^6^ the authors describe the clinical-demographic profile and prognosis of patients with chronic ischemic heart disease (CIHD), followed at a tertiary cardiology care center, the Instituto do Coração (InCor), in São Paulo, Brazil. The registry was prospective, recruited participants between 2016 and 2018, and had a median follow-up time of 2.4 years. Six hundred twenty-five patients were included, but only 533 had follow-up evaluations. The sample had a median age of 66, a third of women, and a high prevalence of morbidities, including 87% of previous cardiovascular events/procedures. The study revealed a small improvement in lipid control during follow-up, especially in LDL cholesterol, but without an apparent correspondence with the prescribed therapy since statins had a similar proportion of prescriptions at the final and baseline moments. The authors draw attention to the low prescription of additional lipid-lowering agents. However, there is no description of the frequency of use of potent statins at the maximum dose, which is recommended before combining other medications. At this point, it would also be important to describe local practice, as the Unified Health System provides simvastatin free of charge, but atorvastatin only through a bureaucratic process, which limits its use. In a public service in the same state, only 3% of outpatient prescriptions were for atorvastatin 80mg/d.^7^

Interpretation of these findings is limited. The study evaluated participants at baseline and each year of follow-up, preferably in face-to-face visits, although these were not guaranteed. Furthermore, there was a high loss to follow-up (11.5%), and there is no description of whether this was associated with risk factors, which may compromise the reliability of information. Furthermore, prescription does not mean treatment adherence.

Regarding clinical outcomes, Moreira et al.^6^ found a 7% incidence of death, MI, or CVA (primary outcome) at the end of follow-up. This finding should be compared to the results of other cohorts and generate reflections, although methodological differences and regional variations may justify differences in the incidence of outcomes. The international multicenter registry CLARIFY^8^ found 8% cardiovascular death or MI (primary outcome) in a 5-year follow-up of patients with CCS. The secondary outcome, composed of cardiovascular death, non-fatal MI, and non-fatal stroke, closer to the primary outcome of study by Moreira et al.^6^, occurred in 9.5%. The CLARIFY cohort evaluated over 30,000 individuals in 45 countries of varying socioeconomic levels. In addition to different follow-up times, the CLARIFY study adjudicated the primary outcome.

In the Brazilian REACT registry (2021), 5076 patients were followed for one year, of which two-thirds were in secondary prevention. The incidence of fatal and non-fatal atherosclerotic cardiovascular events was estimated at 5.46 per 100 patient-years.^9^ The sample profile revealed similarities with the study,^6^ however, methodological differences make comparisons difficult.

Another interesting finding of the study^6^ was the reduction in angina over the 2.4-year follow-up, with an increase in the percentage of asymptomatic patients at the final evaluation.^6^ This data was also found in the CLARIFY registry when evaluating 7212 individuals with CCS and angina pectoris, who underwent optimized medical therapy and showed a 40% reduction in anginal symptoms in one year.^10^ The absence or improvement of angina was associated with a lower incidence of major cardiovascular outcomes, such as death from cardiovascular causes and non-fatal MI,^10^ which supports recommendations for optimized pharmacological therapy as a primary strategy in patients with CCS.^11^

The study^6^has limitations related to convenience sampling, sample size, relatively short follow-up times, and eminently exploratory inferential statistics. Loss to follow-up was high, and the lack of adjudication of events may reduce internal validity. However, the study information is important, contemporary, and useful in characterizing patients with SCC and their medium-term prognosis. It signals the need for similar studies at regional or national multicenter research to obtain information on the effectiveness of therapies and the clinical evolution of patients with SCC.
